# Scholarly literature in HIV-related lesbian, gay, bisexual, and transgender studies: A bibliometric analysis

**DOI:** 10.3389/fpsyg.2023.1028771

**Published:** 2023-02-08

**Authors:** Tham Thi Nguyen, Anh Linh Do, Long Hoang Nguyen, Giang Thu Vu, Vu Anh Trong Dam, Carl A. Latkin, Brian J. Hall, Cyrus S. H. Ho, Melvyn W. B. Zhang, Roger C. M. Ho

**Affiliations:** ^1^Institute for Global Health Innovations, Duy Tan University, Da Nang, Vietnam; ^2^Faculty of Medicine, Duy Tan University, Da Nang, Vietnam; ^3^Institute of Health Economics and Technology, Hanoi, Vietnam; ^4^Department of Global Public Health, Karolinska Institute, Stockholm, Sweden; ^5^National Centre for Youth Substance Use Research, The University of Queensland, Brisbane, QLD, Australia; ^6^Bloomberg School of Public Health, Johns Hopkins University, Baltimore, MD, United States; ^7^School of Global Public Health, New York University, New York, NY, United States; ^8^Department of Psychological Medicine, Yong Loo Lin School of Medicine, National University of Singapore, Singapore, Singapore; ^9^Lee Kong Chian School of Medicine, Nanyang Technological University Singapore, Singapore, Singapore; ^10^Institute for Health Innovation and Technology (iHealthtech), National University of Singapore, Singapore, Singapore

**Keywords:** lesbian, gay, bisexual, transgender, bibliometric, HIV

## Abstract

**Introduction:**

Lesbian, gay, bisexual, and transgendered (LGBT) people are marginalized and understudied. Analyzing research activity worldwide is vital to better understand their needs in confronting the HIV epidemic. This study aimed to evaluate the global literature to identify the research collaboration, content, and tendency in HIV-related issues among the LGBT populations.

**Methods:**

Peer-reviewed original articles and reviews were achieved from the Web of Science Core Collection database. Country’s collaborations and co-occurrence of most frequent terms were illustrated by VOSviewer software. The Latent Dirichlet Allocation (LDA) and the linear regression model were utilized to uncover the hidden topics and examine the research trend.

**Results:**

From 1990 to 2019, a total of 13,096 publications were found. Stigma, sexual risk behaviors and HIV testing were the major topics in the LGBT research during the study period. Among 15 topics, topics about HIV/Sexually transmitted infections (STIs) prevalence, Outcomes of HIV/AIDS care and treatment, and Opportunistic infections in HIV-positive LGBT people showed decreasing attention over years, while other topics had a slight to moderate increase.

**Discussion:**

Our study underlined the exponential growth of publications on the LGBT population in HIV research, and suggested the importance of performing regional collaborations in improving research capacity. Moreover, further research should focus on examining the manner to increase the coverage of HIV testing and treatment, as well as implement HIV-interventions with low cost and easy to scale-up.

## 1. Introduction

The first case of a syndrome now so-called AIDS was presented in 1981 among healthy American men who have sex with men (MSM; CDC, 1981a,b,c). Since then, sexual minorities, including lesbian, gay, bisexual, and transgender (LGBT) groups have been considered among the most vulnerable populations to HIV across nations (Gangamma et al., 2008; Deol and Heath-Toby, 2009; Beyrer et al., 2010, 2012; Marshall et al., 2011). Despite global efforts, mounting evidence in different systematic reviews revealed emerging circumstances of HIV/sexually transmitted infections (STIs) in these marginalized groups. Among MSM, the HIV prevalence was from 3% in the Middle-East and North African countries to 25.4% in Caribbean countries (Beyrer et al., 2012). Another systematic review indicated that in low-and middle-income countries, the HIV prevalence in MSM was 19-fold higher compared with the general population (Baral et al., 2007). The burden of HIV in trans women who have sex with men and sex workers is even more severe, with the prevalence of HIV in these populations approximately 49 times higher compared with the general population at reproductive age (Baral et al., 2013a). Few studies have been conducted to examine the HIV prevalence in sexual minority women, with the range from 1.3% in general women who have sex with women (WSW; Lemp et al., 1995) to 42% among WSW who inject drugs (Diaz et al., 2001).

Although homosexuality is no longer classified as an illness (Association AP, 2013), it does not change the fact that LGBT people are marginalized and often understudied or underrepresented in research and practice (Wolitski et al., 2008; Graham et al., 2011). A recent report of the International Lesbian, Gay, Bisexual, Trans and Intersex Association in 2020 showed that 67/193 countries of the United Nation member states treated same-sex behaviors as illegal acts (Mendos et al., 2020). This criminalization facilitates social stigma or rejection, abuse, and violence against homosexual groups (Dean et al., 2000; Mays et al., 2002; Reisner et al., 2016; Winter et al., 2016a). These issues are related to social isolation, and increased risk of psychological distress and low self-esteem (Díaz et al., 2001), which, consequently, elevate the engagement in the HIV-related risk behaviors (e.g., substance use, condomless use during sexual intercourses or multiple sex partners) and the vulnerability to HIV acquisition in these populations (Hughes and Eliason, 2002; Díaz et al., 2004; McCabe et al., 2009; Evans et al., 2016). Moreover, homophobia may constrain the use of HIV prevention and treatment services and worsen the disparities in healthcare access for the LGBT people (Ayala et al., 2013; Baral et al., 2013b; Pachankis et al., 2015).

Substantial activities to advocate the rights of LGBT populations and alleviate the social stigmatization toward non-heterosexual groups have been developed in the last decades (Health USDo, 2000; Powell et al., 2016; Winter et al., 2016b; Mendos et al., 2020). One important component of these activities is the involvement of academia in research activity to provide scientific evidence regarding LGBT health and human rights, disseminate to the governments and community, and change the social norms about same-sex behaviors. Moreover, the scientific community can inform the healthcare needs of LGBT groups, particularly in responding to the spread of the HIV epidemic (Mayer et al., 2008). To assess the current status of research on LGBT people in the field of HIV/AIDS, a bibliometric study to analyze research activity worldwide is vital to inform a comprehensive overview. Moreover, the findings of this study could be utilized by donors and LGBT activists to advocate researchers to encompass their HIV-related issues in the research agenda as well as diminish the burden of HIV burden in this population. Therefore, this study aimed to evaluate the global literature to identify the research collaboration, content, and themes in HIV-related issues among the LGBT populations.

## 2. Materials and methods

### 2.1. Database, search strategy, and eligible criteria

The Web of Science Core Collection was used to search the publications. This database was selected based on its advantages in providing more comprehensive data compared to other databases such as Scopus or PubMed (Clarivate Analytics, n.d.; Martín-Martín et al., 2018). We set the duration in 20 years from 1990 to 2019, with language restriction to English only. We also included only original articles and reviews which studied or mentioned LGBT population in the field of HIV. Other types of documents were excluded.

In this study, we conducted a search process in two phases. First, we searched the articles about HIV/AIDS by using the topic terms “HIV,” “AIDS,” “Human Immunodeficiency Virus,” and “Acquired Immune Deficiency Syndrome.” Then, with the HIV/AIDS-related dataset, we used the following terms for searching in title/abstract in order to filter studies about the LGBT populations:

1. Terms for lesbian: lesbian, women who have sex with women, WSW, homosexual female, lesbianism
2. Terms for bisexuality: bisexual, bisexuality
3. Terms for transgender: transgender, transgendered, transsexualism, transsexual, gender identity disorders, gender dysphoria
4. Terms for gay: men who have sex with men, MSM, gay
5. General terms: LGBT, homosexuality, homosexual, same-sex, queer, sexual minority, not exclusively heterosexual.

**Figure 1:**
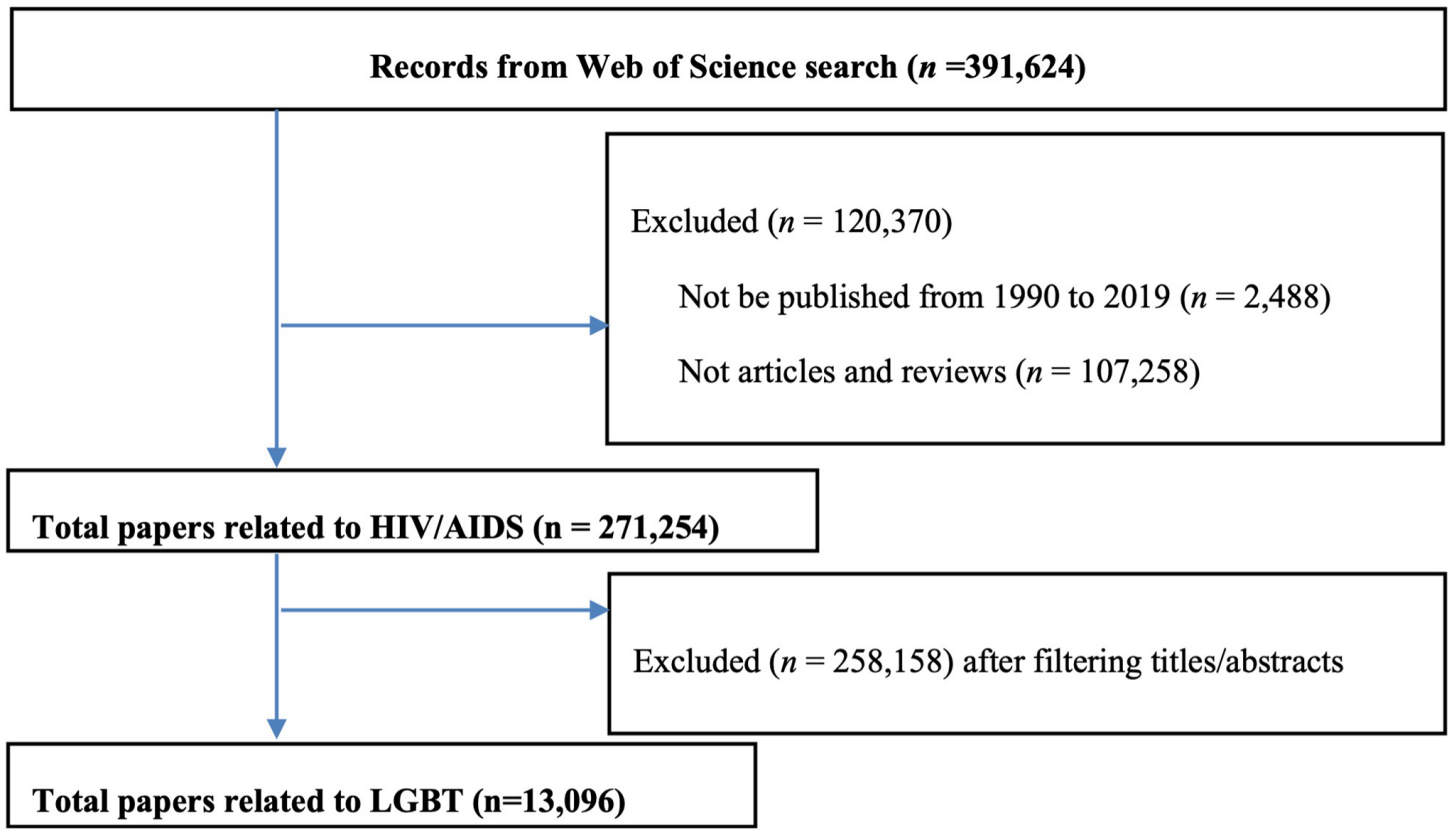
Flow chart of the searching process.

Figure 1 presents the flow chart of the searching process. A total of 13,096 articles were included in the final analysis.

### 2.2. Bibliometric indicators, analysis, and visualization

Information of selected documents was analyzed and presented including the year of publications, the number of publications per year, total citation up to 2019, mean citation rate per year, total usage in the last 6 months/5 years, and mean use rate in the last 6 months/5 years. The VOSviewer (version 1.6.8, Center for Science and Technology, Leiden University, Netherlands) software was employed to visualize the co-occurrence of most frequent terms and the country’s collaboration networks. Stata 15.0 was used for topic modelling and research trend identification.

Latent Dirichlet Allocation (LDA) was employed to examine the contents of 15 latent topics. The LDA is processed based on the text mining principle, which helps to identify the topics among the unstructured body of texts. It is an algorithm that allows grouping a set of words with similar meanings to reflect a specific topic. For example, we assumed that articles on the topic of PWIDs would have the words “injection” or “drug users,” while the words “female,” “sexual,” and “workers” would be appeared more frequently in the articles on the topic of CSW. Topic modelling enables us to define the particular number of topics first, and then extract and allocate the words under these topics. After obtaining the outputs of the LDA model, we labelled the topics by referring two sources: (1) the top 15 words (after sorted) with the highest probability within each topic; and (2) titles/abstracts of publications within each topic. HIV and RDS experts are invited to label the topics in order to provide the most semantically meaningful interpretation.

We also calculate the volume and share of publications per topic per year. A linear regression model was conducted to detect the trend of each research topic in different time periods (1990–2000, 2001–2010, and 2011–2019), with the share of publication as an outcome variable, and the number of years as a predictor variable. “Hot’ topic was defined when the coefficient was significantly positive, and the “cold” topic was detected when the coefficient was significantly negative. Statistical significance was identified if the value of *p* was less than 0.05.

## 3. Results

**Figure 2:**
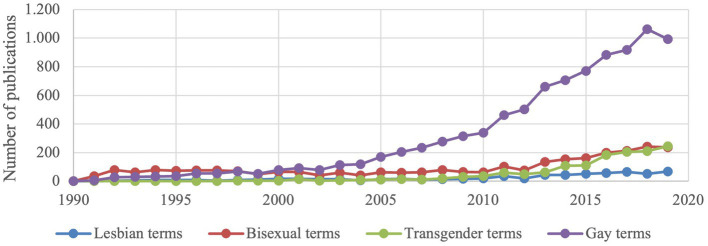
The number of publications by years according to the search terms. Some studies had two or more population.

Table 1 shows the characteristics of the selected articles. From seven articles in 1990, the number of publications remarkably increased to 1,205 in 2019, resulting in a total of 13,096 publications in the whole period. Articles published in 2013 had the highest number of citations (19,584), total usage in the last 5 years (9,873 downloading times), and the mean use rate in the last 5 years (2.57 downloading times/paper/year). The number of publications regarding different populations (lesbian, gay, transgender, and bisexual) is presented in Figure 2. Publications about gay (or men who have sex with men) group had the highest number (accounted for nearly 70% of the total number of publication) over the time period, following by bisexual, transgender, and lesbian.

**Table 1:** General characteristics of publications.

Year published	Total number of papers	Total citations	Mean cite rate per year	Total usage last 6 month	Total usage last 5 years	Mean use rate last 6 month	Mean use rate last 5 year
2019	1,205	1,312	1.09	1,462	2,999	1.21	0.50
2018	1,237	5,505	2.23	773	5,395	0.62	0.87
2017	1,085	9,336	2.87	459	6,737	0.42	1.24
2016	1,041	13,044	3.13	414	8,992	0.40	1.73
2015	904	16,406	3.63	316	9,316	0.35	2.06
2014	826	17,903	3.61	214	8,932	0.26	2.16
2013	768	19,584	3.64	240	9,873	0.31	2.57
2012	585	19,123	4.09	130	6,838	0.22	2.34
2011	561	18,194	3.60	158	5,384	0.28	1.92
2010	425	17,383	4.09	111	3,861	0.26	1.82
2009	394	15,797	3.64	64	2,899	0.16	1.47
2008	353	14,535	3.43	52	2,376	0.15	1.35
2007	316	14,761	3.59	55	1848	0.17	1.17
2006	272	13,120	3.45	53	1,640	0.19	1.21
2005	246	12,315	3.34	32	1,375	0.13	1.12
2004	201	10,323	3.21	21	977	0.10	0.97
2003	202	13,157	3.83	87	1,543	0.43	1.53
2002	156	8,267	2.94	22	672	0.14	0.86
2001	197	12,263	3.28	37	1,088	0.19	1.10
2000	199	8,434	2.12	16	660	0.08	0.66
1999	164	7,030	2.04	22	528	0.13	0.64
1998	212	9,621	2.06	16	528	0.08	0.50
1997	210	8,878	1.84	18	470	0.09	0.45
1996	262	14,847	2.36	26	689	0.10	0.53
1995	222	10,744	1.94	16	393	0.07	0.35
1994	217	8,477	1.50	14	402	0.06	0.37
1993	204	10,572	1.92	15	340	0.07	0.33
1992	237	9,946	1.50	17	345	0.07	0.29
1991	188	8,850	1.62	9	359	0.05	0.38
1990	7	315	1.50	2	57	0.29	1.63

**Figure 3:**
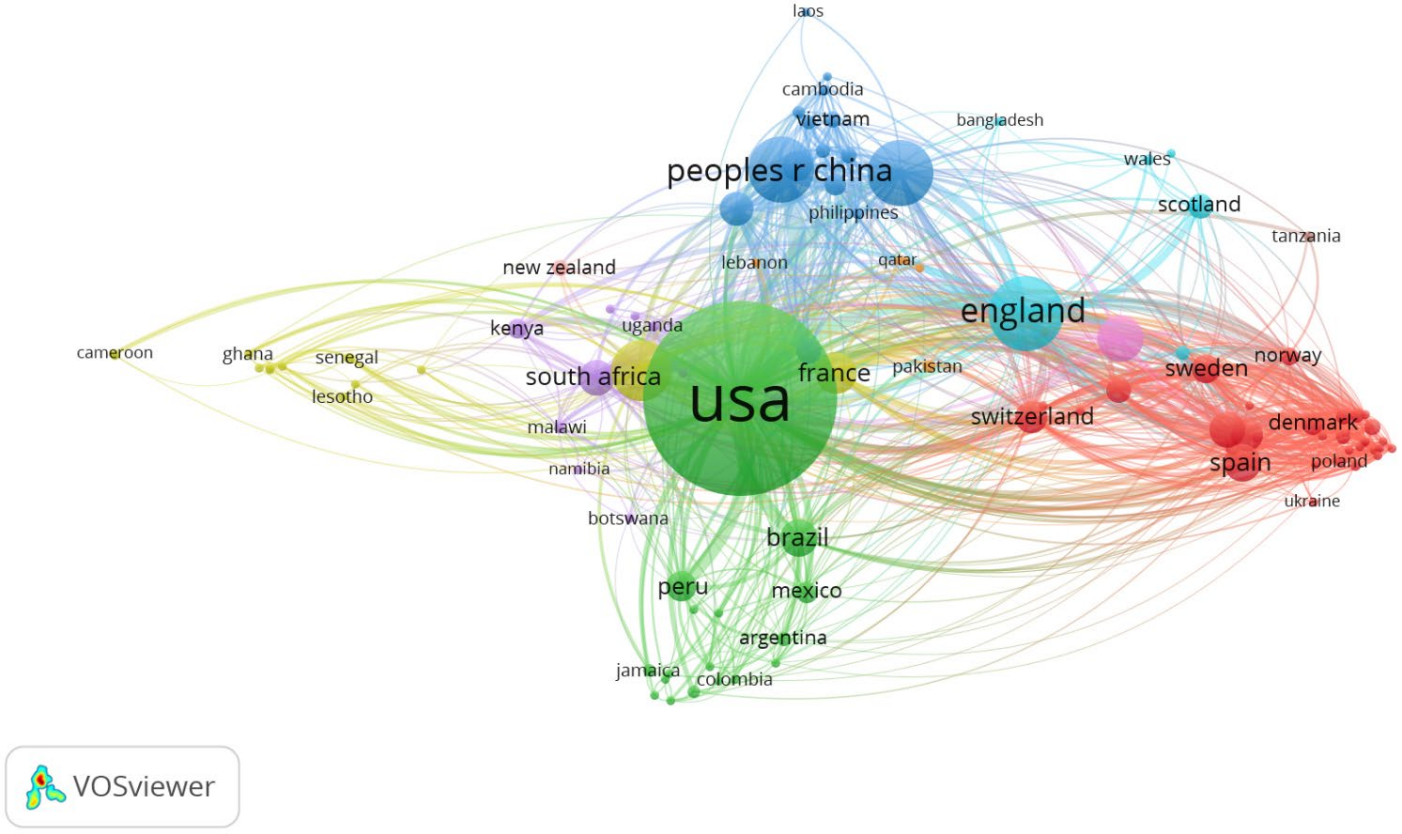
Collaborations between countries.

Figure 3 illustrates the collaborations among countries in HIV-related LGBT research. Publications in this field were produced by authors from 156 countries from 1990 to 2019, of which 107 countries had five publications or more. There were 11 clusters of country collaborations. The top five largest clusters were (1) red cluster (with European countries, except France), (2) green cluster (with North and South American countries, except Canada), (3) blue cluster (with East and Southeast Asian countries), (4) yellow cluster (with North African countries, including Canada and France), and (5) purple cluster (with South African countries). These clusters were led by Spain (301 publications), the United States (7,719 publications), China (916 publications), Canada (745 publications), and South Africa (267 publications), respectively.

Table 2 presents the top 20 publications with the highest citations. Most of the studies published before 2000 were about molecular epidemiology of HIV and Kaposi’s-Sarcoma, while the studies published after this time had a variety of topics such as STIs, HIV incidence, mental health, and pre-exposure prophylaxis, with different study designs including cross-sectional, randomized controlled trials and reviews. The first article entitled “Prejudice, social stress, and mental health in lesbian, gay, and bisexual populations: Conceptual issues and research evidence” by Ilan H. Meyer presented the prevalence of mental disorders among LGB populations (Meyer, 2003). The second publication entitled “Preexposure Chemoprophylaxis for HIV Prevention in Men Who Have Sex with Men” conducted by Grant et al. was the randomized controlled trial to examine the effectiveness of pre-exposure prophylaxis [emtricitabine and tenofovir disoproxil fumarate (FTC–TDF)] in HIV prevention among men and transgender women who have sex with men (Grant et al., 2010). The third study was “Sexually Transmitted Diseases Treatment Guidelines, 2015” from the United States Center for Disease Prevention and Control (CDC), which updated the treatment for different STIs, especially for transgender people (Workowski and Bolan, 2015).

**Table 2:** Top 20 most cited papers.

No	Title	Journal	Cite	Year	Cite rate
1	Prejudice, social stress, and mental health in lesbian, gay, and bisexual populations: Conceptual issues and research evidence	Psychological Bulletin	3,846	2003	226.2
2	Preexposure Chemoprophylaxis for HIV Prevention in Men Who Have Sex with Men.	New England Journal Of Medicine	2,601	2010	260.1
3	Sexually Transmitted Diseases Treatment Guidelines, 2015	MMWR Recommendations And Reports	1,234	2015	246.8
4	The role of a mutant CCR5 allele in HIV-1 transmission and disease progression	Nature Medicine	1,039	1996	43.3
5	Estimation of HIV incidence in the United States	JAMA-Journal Of The American Medical Association	983	2008	81.9
6	Detection of herpesvirus-like DNA-sequences in Kaposi’s-sarcoma in patients with and those without HIV-infection	New England Journal Of Medicine	961	1995	38.4
7	Lytic growth of Kaposi’s sarcoma-associated herpesvirus (human herpesvirus 8) in culture	Nature Medicine	832	1996	34.7
8	Quantitation of HIV-1 RNA in plasma predicts outcome after seroconversion	Annals Of Internal Medicine	809	1995	32.4
9	Herpes simplex virus 2 infection increases HIV acquisition in men and women: systematic review and meta-analysis of longitudinal studies	AIDS	802	2006	57.3
10	Global epidemiology of HIV infection in men who have sex with men	Lancet	795	2012	99.4
11	Influence of combinations of human major histocompatibility complex genes on the course of HIV-1 infection	Nature Medicine	766	1996	31.9
12	Primary effusion lymphoma: A distinct clinicopathologic entity associated with the Kaposi’s sarcoma-associated herpes virus	Blood	751	1996	31.3
13	Prognostic value of HIV-1 syncytium-inducing phenotype for rate of Cd4+ cell depletion and progression to AIDS	Annals Of Internal Medicine	740	1993	27.4
14	Estimated HIV Incidence in the United States, 2006–2009	PloS One	712	2011	79.1
15	A difference in hypothalamic structure between heterosexual and homosexual men	Science	691	1991	23.8
16	Pre-exposure prophylaxis to prevent the acquisition of HIV-1 infection (PROUD): effectiveness results from the pilot phase of a pragmatic open-label randomized trial	Lancet	668	2016	167.0
17	The seroepidemiology of human herpesvirus 8 (Kaposi’s sarcoma-associated herpesvirus): Distribution of infection in KS risk groups and evidence for sexual transmission	Nature Medicine	602	1996	25.1
18	Association of co-occurring psychosocial health problems and increased vulnerability to HIV/AIDS among urban men who have sex with men	American Journal Of Public Health	586	2003	34.5
19	On-demand preexposure prophylaxis in men at high risk for HIV-1 infection	New England Journal Of Medicine	583	2015	116.6
20	Sexual behaviour in Britain: partnerships, practices, and HIV risk behaviours	Lancet	577	2001	30.4

**Figure 4:**
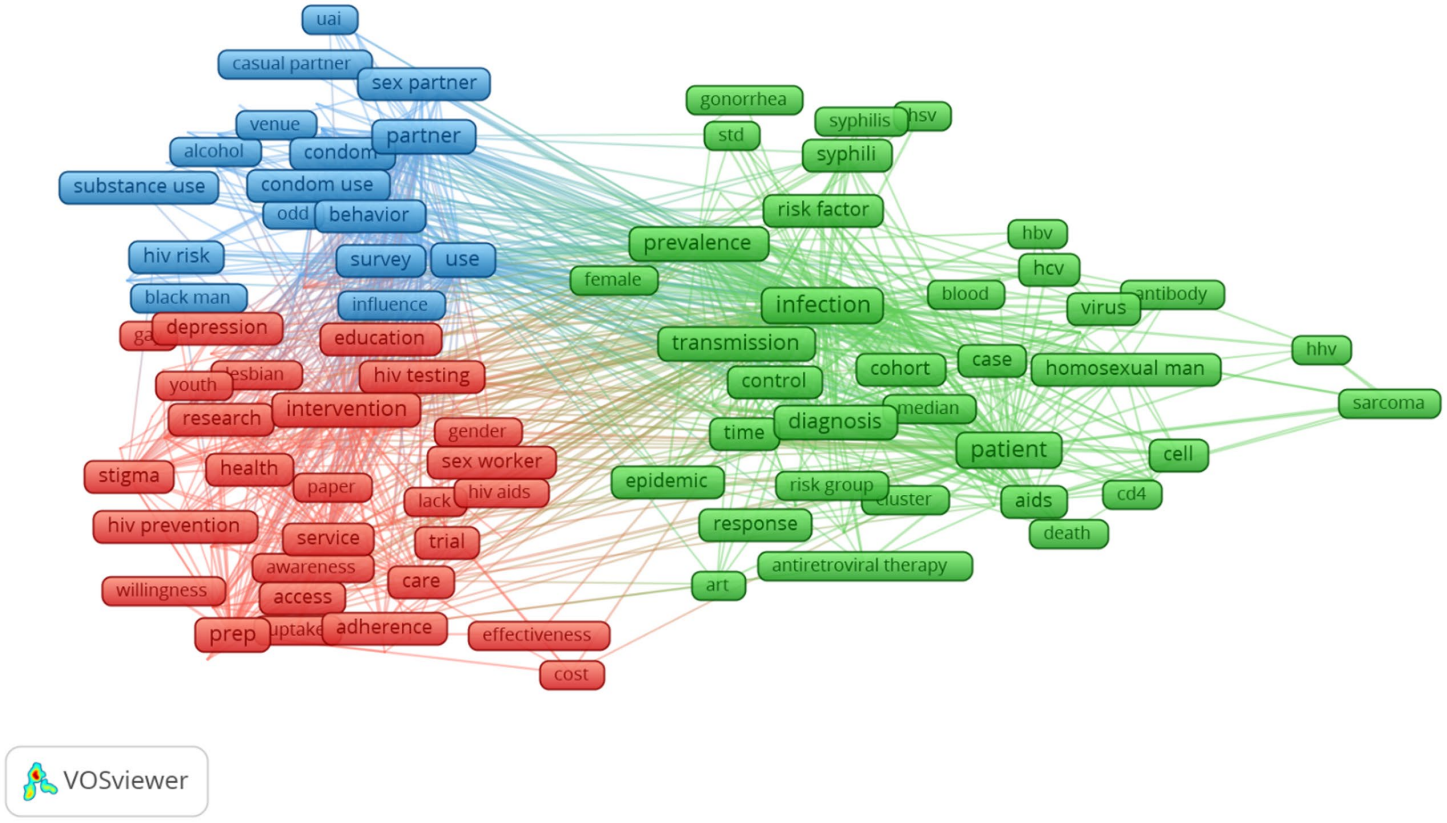
Co-occurrence of most frequent terms in titles and abstracts.

Analysis of the most frequent terms is visualized in Figure 4. A total of three clustering of co-occurrence terms were developed. The green cluster mentioned topics about HIV/STIs prevalence, risk factors, and treatment among LGBT populations. Meanwhile, the blue cluster referred to topics about HIV-related risk behaviors (substance use, sexual risk behaviors), and the red cluster indicated topics about HIV prevention and testing service access (including HIV testing, pre-exposure prophylaxis), stigma and mental health.

**Figure 5:**
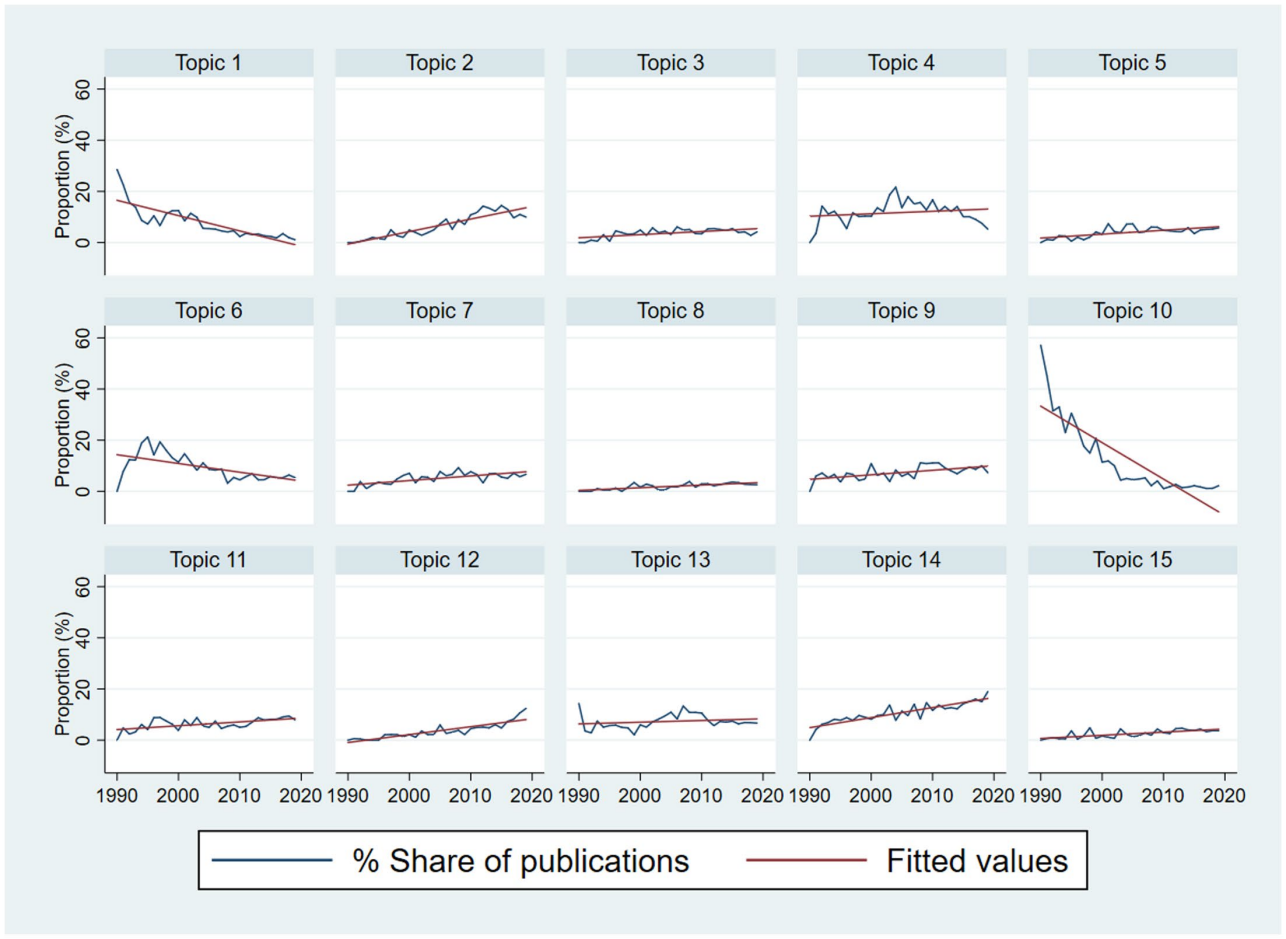
Trends in research topics by year during 1990–2019.

Results of topic modelling by using the LDA technique are revealed in Table 3. Three topics with the highest number of publications were (1) Topic 14: “Stigma-related barriers in health communication and health care access” (11.6%); (2) Topic 4 “Sexual risk behaviors and associated factors” (9.9%), and (3) Topic 2 “HIV testing and associated factors” (8.3%). The trend of research topics *via* publication share (%) is visualized in Figure 5. Data on topics 1, 6, and 10 presented a downward trend over the years, while other topics had a slight to moderate increase over the period.

**Table 3:** Fifteen topics classified by LDA and 15 most frequent terms in each topic.

No	Topic	Most frequent terms	n	%
1	Stigma-related barriers in health communication and health care access	Health; social; prevention; research; services; transgender; community; sexual; stigma; people; study; qualitative; barriers; access; interviews	1,519	11.6%
2	Sexual risk behaviors and associated factors	Sexual; partners; partner; condom; intercourse; unprotected; reported; behavior; behaviors; associated; study; bisexual; between; HIV-positive; transmission	1,297	9.9%
3	HIV testing and associated factors	Testing; prevalence; study; associated; china; participants; survey; tested; results; infection; having; factors; methods; reported; months	1,090	8.3%
4	HIV epidemic modeling and resource allocation for HIV prevention, treatment and care	Studies; population; prevention; epidemic; populations; review; prevalence; transmission; incidence; countries; model; infections; treatment; infection; interventions	961	7.3%
5	Outcomes of HIV/AIDS care and treatment	Patients; cohort; incidence; study; years; associated; viral; HIV-infected; antiretroviral; diagnosis; therapy; infection; between; treatment; count	872	6.7%
6	Mental health and coping strategies	Health; sexual; associated; depression; social; between; stigma; study; support; mental; symptoms; psychosocial; abuse; psychological; relationship	855	6.5%
7	Sexually transmitted infections (STIs) testing, diagnose and treatment	Syphilis; infection; infections; sexually; testing; patients; transmitted; clinic; diagnosed; screening; cases; diagnosis; results; chlamydia; health	844	6.4%
8	Opportunistic infections in HIV-positive LGBT	Patients; homosexual; infection; disease; immunodeficiency; cells; clinical; human; patient; cases; virus; sarcoma; immune; subjects; Kaposi’s	723	5.5%
9	Inequalities of HIV-related risk behaviors by gender, race, ethnicity and sexual orientation	Women; sexual; black; transgender; young; health; heterosexual; states; reported; bisexual; behaviors; African; united; American; white	673	5.1%
10	Feasibility, acceptability and efficacy of pre-exposure prophylaxis interventions	Intervention; participants; prophylaxis; adherence; pre-exposure; prevention; study; trial; efficacy; interventions; trials; acceptability; randomized; group; follow-up	668	5.1%
11	HIV/Sexually transmitted infections (STIs) prevalence	Infection; virus; hepatitis; human; immunodeficiency; prevalence; blood; patients; homosexual; antibody; antibodies; positive; seroprevalence; HIV-1; subjects	544	4.2%
12	Substance use, associated factors and related to risky sexual behaviors	Associated; alcohol; substance; sexual; drugs; methamphetamine; users; reported; behaviors; study; factors; between; participants; prevalence; using	534	4.1%
13	Molecular epidemiology of different types of HIV	HIV-1; transmission; subtype; resistance; sequences; infected; analysis; individuals; strains; clusters; china; identified; patients; epidemic; phylogenetic	476	3.6%
14	Human papillomavirus infection and vaccination	Cancer; human; HIV-positive; infection; screening; vaccination; papillomavirus; prevalence; vaccine; cytology; intraepithelial; results; lesions; HIV-negative; study	374	2.9%
15	Socio-legal aspects of homosexuality	Sexual; article; religious; queer; sexuality; HIV/aids; same-sex; homosexuality; identity; gender; hombres; cultural; power; masculinity; hommes	285	2.2%

In the 90s decade, the topics about HIV testing (topic 2), molecular epidemiology of different types of HIV (topic 3), substance use (topic 5), inequalities in HIV-related risk behaviors (topic 7), socio-legal aspects of homosexuality (topic 8), pre-exposure prophylaxis (topic 12), and stigma-related barriers (topic 14) showed the positive trend, while the topics about HIV/STI prevalence (topic 1), and opportunistic infections (topic 10) had the negative tendency. From 2001 to 2010, topics 2 and 7 remained the positive trend, along with topic 9 (resource allocation) and topic 13 (STIs testing, diagnoses, and treatment); whereas the publication share of topic 1, topic 6 (HIV treatment outcomes), and topic 10 continuously decreased over this period. During 2011–2019, topics 1, 3, and 4 (sexual risk behavior) became “cold” topics, while topics 11 (mental health), 12, and 14 were more popular. Overall, topics 1, 6, 10 showed a negative trend, while topics 2, 3, 5, 7, 8, 9, 11, 12, 14, and 15 showed a positive trend (Table 4).

**Table 4:** Hot/Cold topics.

Topic	1990–2000		2001–2010		2011–2019		Overall	
	Coefficient	Hot/Cold	Coefficient	Hot/Cold	Coefficient	Hot/Cold	Coefficient	Hot/Cold
1	−1.34*	Cold	−0.81*	Cold	−0.23*	Cold	−0.60*	Cold
2	0.42*	Hot	0.72*	Hot	−0.39	-	0.49*	Hot
3	0.48*	Hot	0.01	-	−0.26	Cold	0.12	Hot
4	0.55	-	0.01	-	−0.95*	Cold	0.10	-
5	0.26*	Hot	−0.08	-	0.14	-	0.15	Hot
6	0.90	-	−1.01*	Cold	−0.01	-	−0.34*	Cold
7	0.61*	Hot	0.41*	Hot	0.10	-	0.18*	Hot
8	0.23*	Hot	0.12	-	0.01	-	0.10*	Hot
9	0.40	-	0.59*	Hot	−0.15	-	0.18*	Hot
10	−3.68*	Cold	−0.90*	Cold	−0.06	-	−1.43*	Cold
11	0.49	-	−0.25	-	0.31*	Hot	0.15*	Hot
12	0.23*	Hot	0.17	-	0.91*	Hot	0.31*	Hot
13	−0.42	-	0.59*	Hot	−0.04	-	0.07	-
14	0.66*	Hot	0.25	-	0.64*	Hot	0.39*	Hot
15	0.20	-	0.19	-	−0.004	-	0.12*	Hot

* *p*-value < 0.0.

## 4. Discussion

This study evaluated the development of publications on the LGBT populations in the field of HIV/AIDS in the period from 1990 to 2019. Study findings indicated remarkable research productivity in this area over time, which focused mainly on gay or men who have sex with men groups, with the major topics about HIV-related risk behaviors, prevention and treatment service access, stigma, and mental health. Moreover, by analyzing the research topics, we found the change of research focus in the different time intervals, which can be useful for proposing future research agenda.

Our analysis indicated the rapid expansion of publications studying HIV-related issues in the LGBT groups from the early 90s, one decade after the first AIDS case was detected (CDC, 1981b). This substantial growth of publications was mainly driven by the United States, England, China, Canada, and Australia. They were also among the top contributors to the HIV research in other aspects such as antiretroviral therapy (Tran et al., 2020a), behavioral health interventions (Tran et al., 2019a), pre-exposure prophylaxis (Tran et al., 2020b), or economic evaluation studies (Tran et al., 2019b). Our result was similar to the previous bibliometric analysis among transgender people (Sweileh, 2018). However, the finding suggested that the contributions to the LGBT publications were skewed to North American, European, East and Southeast Asian countries. It is not surprising that they had high research productivity because the LGBT populations are increasingly accepted in these nations (Flores, 2019). The number of publications was sparse among African and Mediterranean countries, particularly in Islamic states such as Qatar, Egypt, and others. This low productivity may be due to low research capacity, a low number of LGBT people, or the cultural/legal context that did not permit same-sex behaviors (Ilkkaracan, 2015; Gökengin et al., 2016; Kaplan and El Khoury, 2017; Mendos et al., 2020). Notably, we found that the countries tended to collaborate with others in the same region. The reason could be the convenience in implementing research when the researchers were more likely to collaborate with others who were geographically close to them. In addition, this finding might suggest the cultural sensitivity of LGBT research. For example, Staples et al. in their qualitative research found that problems about cultural sensitivity should be critically considered when performing research on transgendered people (Staples et al., 2018). Moreover, each nation had its own manners in confronting the LGBT’s problems, including laws, rights, or disease patterns. Therefore, it requires the needs for strong research hubs in different regions, with the country with the highest productivity as a central, to boost the research capacity of country members in the hubs.

By analyzing the growth of publications in different groups, we found that research on gay or MSM outweighed other populations, or in other words, global scholars paid more attention to MSM compared with lesbian, bisexual, or transgender people. This finding is understandable since the first AIDS record was related to MSM, and they are also one of the most-at-risk population for HIV infection due to unprotected anal sex practice, multiple sex partners, high frequency of substance use, large sexual network size, and highly stigmatized (Beyrer et al., 2012; Evans et al., 2016). In terms of transgender people, previous reviews showed a lack of data about HIV/STIs prevalence, health status, and healthcare access (Baral et al., 2013a; Vaitses Fontanari et al., 2019). However, given the matter that our analysis only included studies published in 2019 backward, and more research on this population could be done from 2020 until now, the knowledge gaps in transgendered people might be significantly fulfilled. In addition, data showed that bisexual people were understudied. This phenomenon could be explained by the matter that bisexual population was mostly studied under the term “men who have sex with men” and not directly mentioned with the term “bisexual” in the title/abstract of the publication. Therefore, our text mining technique could not differentiate studies with exclusive MSM and MSM who also have sex with women. It was the limitation of this study that should be addressed in further research. Meanwhile, in lesbian and bisexual women groups, the number of publications was low which might be due to that in these populations, global research prioritized other health problems such as mental health, lung or breast cancers, or other sexually transmitted infections instead of HIV/AIDS (Roberts, 2001; Institute of Medicine Committee on Lesbian GB et al., 2011). However, a prior research by Teti et al. found that non-heterosexually identified women had a higher likelihood of reporting HIV-related risk behaviors such as selling drugs or sex works than heterosexual women (Teti et al., 2007). Many of them rejected or ignored protective behaviors, as well as not being included in the HIV prevention interventions (Richardson, 2000; Teti et al., 2007). It is suggested that more studies should be performed in order to enhance knowledge about HIV/AIDS in these populations and improve their accessibility to HIV/AIDS prevention and care.

The current study indicated that stigma, sexual risk behaviors, and HIV testing were the major topics in LGBT research during 1990–2019 with the highest number of publications. This finding was different from the previous bibliometric study, which found that mental health was the dominant topic in the field of the transgender population (Sweileh, 2018). This difference can be explained by the fact that the authors detected the main topics *via* articles with the most citations; whereas our advantage is the application of LDA, an unsupervised machine learning technique, to take into account all 13,096 papers to explore latent topics. Our results were also different from a bibliometric analysis in general LGBT studies, which found that social sciences were the main research topics, following by medicine and law (Antell, 2012). This dissimilarity was due to the different study scopes as our study focused on LGBT studies in HIV research.

Moreover, we examined the research trend in the whole period by measuring “hot” and “cold” topics, which might support to identify which areas should be prioritized in the future research and policy agenda. We found that in the period 1991–2000, there was an upward trend of topics regarding HIV testing, HIV molecular epidemiology, social inequalities in HIV-related risk behaviors, socio-legal perspectives of same-sex practice, HIV pre-exposure prophylaxis and stigma barriers to healthcare access. Several reasons may explain the blooming of topics of interest in the 1990s. First, during this period, research on HIV/AIDS began to be more performed and funded than it was before 1990. In particular, in the United States, the Ryan White HIV/AIDS Program law was enacted in 1990 as a measure to help reduce the growing burden of HIV/AIDS for the community (Institute of Medicine, 2004). The LGBT community also benefited from this transformation, including an increase in research and the establishments of community organizations supporting the LGBT community in HIV/AIDS prevention and care. Second, numerous activities were performed in this period to advocate the right of LGBT groups in healthcare needs and access (Mayer et al., 2008). Third, publications in these areas addressed the important questions about effective interventions and the target populations of focus. Increasing the awareness of these problems could be served as the foundation for the scale-up of HIV/AIDS interventions in the next decades. As such, from 2001 to 2010, the research focus, while remaining the attention in HIV testing and social inequalities, shifted to resource allocation and STI testing and treatment in the LGBT populations. This period observed an increasing expansion of HIV/AIDS prevention and treatment programs across countries with financial support from foreign donors such as the Global Fund, The U.S. President’s Emergency Plan for AIDS Relief (PEPFAR), or Bill & Melinda Gate Foundation (UNAIDS, 2017). Of note, the LGBT people were among the main target populations for interventions along with injection drug users and commercial sex workers (Organization WH, 2014). A priority research question in this period was the methods to allocate constrained resources optimally and boost the accessibility of HIV/STI testing and treatment services. In the third period from 2011 to 2019, when foreign aid was rapidly reduced and the standard guidelines for HIV prevention and treatment were published (Organization WH, 2013, 2014, 2015, 2017), the attention of scholars transferred to address the remaining issues such as stigma and mental health (Ayala and Santos, 2016; Vaitses Fontanari et al., 2019), and test the novel preventive therapies such as pre-exposure prophylaxis (Tran et al., 2020b) and vaccine (Chuang et al., 2019). Collectively, the research priorities in different periods reflected the changes in the view of communities to the LGBT population, from disease prevention, stigma improvement, to the ultimate goals as LGBT people’s quality of life enhancement. In the upcoming decades, studies that help to increase the coverage of HIV testing and treatment, as well as implement HIV-interventions with low cost and ease to scale-up such as internet-based interventions, which has shown promise in HIV prevention and treatment in homosexual populations (Nguyen et al., 2019), can become the priorities in the research agenda.

Limitations of this study should be noted. First, we did not cover all types of documents, which might underestimate the publications regarding minority groups such as lesbian, bisexual, or transgendered people. Moreover, our analysis only included publications from 1990 to 2019, and did not cover publications from 2020 onward. Further studies with updated data from 2020 until present should be performed to measure the global research trend on LGBT population, particularly after the pandemics such as COVID-19 or Human Monkeypox. Second, we selected only original articles and reviews with English restriction, which might limit the publications from non-native English countries. Given the cultural sensitivity of the research problems regarding LGBT population, other studies should be performed to examine non-English databases of scientific articles. Finally, our topic modelling was performed *via* title/abstract rather than the full-text of publications.

## 5. Conclusion

Our study underlined the exponential growth of publications on the LGBT population in HIV research and the needs of more empirical data on lesbian, bisexual, and transgender people. The finding suggested the importance of performing regional collaborations in improving research capacity. Moreover, further research should focus on examining the manner to increase the coverage of HIV testing and treatment, as well as implement HIV-interventions with low cost and easy to scale-up.

## Data availability statement

The original contributions presented in the study are included in the article/supplementary material, further inquiries can be directed to the corresponding author.

## Ethics statement

Ethical review and approval was not required for the study on human participants in accordance with the local legislation and institutional requirements. Written informed consent for participation was not required for this study in accordance with the national legislation and the institutional requirements.

## Author contributions

TN, LN, GV, and RH: conceptualization. AD, VD, BH, and MZ: data curation. AD, VD, CL, and BH: formal analysis. GV, CL, CH, and MZ: investigation. LN, VD, and BH: methodology. CL, CH, and MZ: supervision. TN, LN, VD, CL, and RH: writing—original draft. TN, AD, LN, GV, CH, and RH: writing—review and editing. All authors contributed to the article and approved the submitted version.

## Funding

The article process charge of this paper is supported by NUS Department of Psychological Medicine (R-177-000-100-001/R-177-000-003-001/R177000702733) and NUS iHeathtech Other Operating Expenses (R-722-000-004-731).

## Conflict of interest

The authors declare that the research was conducted in the absence of any commercial or financial relationships that could be construed as a potential conflict of interest.

## Publisher’s note

All claims expressed in this article are solely those of the authors and do not necessarily represent those of their affiliated organizations, or those of the publisher, the editors and the reviewers. Any product that may be evaluated in this article, or claim that may be made by its manufacturer, is not guaranteed or endorsed by the publisher.
